# Prevalence and Phylogenetic Analysis of Parvovirus (B19V) among Blood Donors with Different Nationalities Residing in Qatar

**DOI:** 10.3390/v13040540

**Published:** 2021-03-24

**Authors:** Doua Abdelrahman, Duaa W. Al-Sadeq, Maria K. Smatti, Sara A. Taleb, Raed O AbuOdeh, Enas S. Al-Absi, Asmaa A. Al-Thani, Peter. V. Coyle, Nader Al-Dewik, Ahmed A. Al Qahtani, Hadi M. Yassine, Gheyath K. Nasrallah

**Affiliations:** 1 Department of Human Genetics, Sidra Medicine Doha, Doha, Qatar; dabdelrahman@sidra.org; 2 Biomedical Research Center, Member of QU Health, Qatar University, Doha, Qatar; da1206066@qu.edu.qa (D.W.A.-S.); msmatti@qu.edu.qa (M.K.S.); enas.alabsi@qu.edu.qa (E.S.A.-A.); aaja@qu.edu.qa (A.A.A.-T.); hyassine@qu.edu.qa (H.M.Y.); 3 College of Medicine, Member of QU Health, Qatar University, Doha, Qatar; 4 College of Health and Life Sciences, Hamad Bin Khalifa University, Doha, Qatar; staleb@hbku.edu.qa; 5 Medical Laboratory Sciences Department, College of Health Sciences, University of Sharjah, Sharjah, United Arab Emirates; rabuodeh@sharjah.ac.ae; 6 Department of Biomedical Science, College of Health Sciences, Member of QU Health, Qatar University, Doha, Qatar; 7 Division of Virology, Department of Pathology and Laboratory Medicine, Hamad Medical Corporation, Doha, Qatar; pcoyle@hamad.qa; 8 Department of Pediatrics, Clinical and Metabolic Genetics, Hamad Medical Corporation, Doha, Qatar; naldewik@hamad.qa; 9 Department of Infection and Immunity, Research Center, King Faisal Specialist Hospital and Research Center, Riyadh 11564, Saudi Arabia; aqahtani@kfshrc.edu.sa; 10 Department of Microbiology and Immunology, Alfaisal University School of Medicine, Riyadh 11533, Saudi Arabia

**Keywords:** B19V, seroprevalence, blood donors, viremia, transfusion

## Abstract

Human parvovirus (B19V) is the causative agent of erythema infectiosum in children and is linked to a wide range of clinical manifestations. Studies related to B19V prevalence in the Middle East and North Africa (MENA) region and other parts of Asia are very scarce. The objectives of this study were to estimate the seroprevalence (anti-B19V IgM and IgG), the viremia rate (B19V DNA), and the circulating genotypes of B19V among blood donors in Qatar. Methods: Donors’ blood samples (*n* = 5026) from different nationalities, mainly from the MENA region and South East Asia, were collected from 2014–2016. Samples were tested for the B19V DNA using RT-PCR. Furthermore, 1000 selected samples were tested to determine the seroprevalence of B19V antibodies using enzyme-linked immunosorbent assay (ELISA). Genotyping was performed on 65 DNA positive samples by sequencing of nested PCR fragments (NS1-VP1u region, 927 nt). Results: Only 1.4% (70/5026) of the samples had detectible B19V DNA in their blood. B19V DNA prevalence statistically decreased with age (*p* = 0.03). Anti-B19V IgG was detected in 60.3% (561/930) of the tested samples, while only 2.1% (20/930) were IgM-positive and 1.2% (11/930) were both IgM- and IgG-positive. B19V genotyping showed a predominance of Genotype 1 (100%). Sequence analysis of the NS1-VP1u region revealed 139 mutation sites, some of which were amino acid substitutions. Conclusion: Our results indicated a relatively high seroprevalence of B19V in Qatar. Most importantly, B19 DNA was detected among Qatari and non-Qatari blood donors. Therefore, blood banks in Qatar might need to consider screening for B19V, especially when transfusion is intended for high-risk populations, including immunocompromised patients.

## 1. Introduction

Human parvovirus (B19V) is a nonenveloped human virus that belongs to the genus *Erythrovirus* of the family *Parvoviridae* family [1]. It infects humans and replicates in the nucleus of erythroid progenitor cells, leading to impaired erythropoiesis [2,3]. Consequently, B19V is the causative agent of erythema infectiosum (fifth disease) or what is known as slapped cheek syndrome [4]. Although the virus mostly infects infants and children, resulting in no or mild disease, it can also infect adults, particularly those with confounding health conditions [5]. B19V has a single-stranded DNA genome that codes for five proteins using the same promoter, p6 [1,6]. The B19V genome contains two main open reading frames (ORFs), which play a vital role in viral DNA replication and transcription, as well as encoding the major and minor structural capsid proteins.

There are three distinct B19V genotypes: Genotype 1 (B19V-related viruses), Genotype 2 (A6-related viruses), and Genotype 3 (V9-related viruses) [7]. These genotypes are distinguished by differences in the coding genes NS1, VP1, VP2, and VP1u [8,9,10,11]; they differ by 10% in the genomic coding region and 20% in the promoter area [7,12,13]. B19V Genotype 1 can be further subdivided into the predominant 1A and the rarely found 1B, while genotype-3 is subdivided into B19/3A and B19/3B [14,15,16,17]. This classification is based on the genetic diversity of the NS1-VP1u region, which shows a higher degree of polymorphism in the VP1u region [8].

The transmission of B19V is primarily through the upper respiratory route, although other routes, such as organ transplantation and blood transfusion, have also been reported [18]. The risk of B19V infection from single-donor blood products is extremely low. However, since large numbers of blood donations make up the plasma pools used to produce plasma derivatives, such as concentrated clotting factors, these products could be frequently contaminated with B19V due to its resistance to most viral inactivation methods [19,20]. Blood banks rely on heat and solvent detergent treatments to ensure the safety of blood products [21,22]. Although these procedures can markedly reduce the B19V DNA viral load, it has been found that B19V can still be detected in treated plasma batches [19,23]. In addition, heat and solvent detergents do not entirely inactivate B19V, which might pose a potential hazard for blood product recipients [24,25,26,27,28,29].

B19V infection is more deleterious in the high-risk population who are frequently multi-transfused. These include immunocompromised patients, especially those with hematological disorders, and pregnant women, particularly in the first and second trimesters [30]. It is estimated that 30–75% of the world’s population is B19V seropositive due to infection during childhood [31,32,33,34]. Although the prevalence of B19V DNA in asymptomatic blood donors’ plasma is very low, a few reports, particularly in developing countries, indicated that B19V prevalence could approach 1.3% [17,31,32,34,35,36,37]. To the best of our knowledge, no studies were conducted in Qatar nor in the Middle East and North Africa (MENA) region on B19V detection and genotyping in healthy donors with large sample size, as in our study. Additionally, Qatar is a country with a diverse population in which expatriates constitute more than 85% [38]. Therefore, this study aimed to estimate the rate of B19V infection among healthy blood donors in Qatar and determine the demographic distribution of genotypes and sub-genotypes in relation to gender, ethnicity, and age. The information gained will enable the health stakeholders in Qatar to develop new guidelines aiming to reduce the burden of transmissible diseases related to blood transfusion.

## 2. Materials and Methods

### 2.1. Ethical Approval and Sample Collection

A total of 5026 blood samples were anonymously collected from blood donors over a period of 3 years (2014–2016), along with all necessary demographic information including age, nationality, and gender. Blood samples used in this study were previously used in other research studies [39,40,41,42,43]. Sample collection was approved by the Hamad Medical Corporation (HMC) Institutional Review Board (HMC-IRB-14292/14) and Qatar University Institutional Review Board (IRB) (QU-IRB 867-E/18). The demographic characteristics of the donors are summarized in Table 1 and Table 2.

### 2.2. DNA Extraction

All 5026 participants’ plasma was tested in pools of 10 plasma samples (400 μL). The DNA-positive pools were further subjected to individual DNA extraction using the Qiagen kit (Catalog #51106, Qiagen, Hilden, Germany) as per the manufacturer’s instructions. The purity and the concentration of all extracted DNA samples were measured using the NanoQuant microplate reader (Infinite Pro200, Tecan, Männedorf, Switzerland). The extracted DNA samples were stored at –20 °C for further testing.

### 2.3. B19V DNA Detection by Quantitative Real-Time PCR

Detection of positive samples from the extracted DNA pools was performed using a real-time PCR detection and quantification kit (Catalog #TV49-50FRT, Sacace, Como, Italy) according to the manufacturer’s instructions. Subsequently, samples from positive pools were subjected to individual detection and quantification of B19V-DNA using the same kit. The detection principle of this assay was based on using real-time amplification with fluorescent reporter dye probes specific for the B19V VP1 gene. The fluorescent dyes were detected by the QuantStudio 6 Flex Real-Time PCR reader (Applied Biosystems, Waltham, MA, USA). The kit included an indigenous internal control (IC) to detect any possible reaction inhibition. The reaction was considered valid only if the quantity of the IC was more than 1.65 × 10^5^ copies per reaction and no amplification was detected in the negative control. B19V viral load was calculated in copies per reaction and copies per mL.

### 2.4. Qualitative ELISA Testing of B19V Antibodies

After identifying the B19V DNA-positive samples, we were interested in determining the immune status and the serological profile of these samples. An additional 930 randomly selected samples were chosen based on area or region. Estimating the seroprevalence of B19V antibodies was accomplished by the screening these plasma samples using commercial qualitative ELISA kits (NovoLisa TM, Parvovirus B19-ELISA; Novatec Immunodiagnostic GmbH, Dietzenbach, Germany), which included screening antibodies specific for VP1 and VP2 antigens (Immunoglobulin G (IgG) (Catalog #PARG0370) and Immunoglobulin M (IgM) (Catalog #PARM0370)) following the manufacturer’s recommendations. Samples were considered positive if the absorbance value was above the cut-off value and were retested when the value was equivocal.

### 2.5. Immune Status Classification of Blood Donors

The immune status of the B19V DNA-positive samples (acute infection, past infection, persistent infection, and reactivated infections) was classified according to the literature [44,45,46] (Table 1). The presence of B19V DNA only, or B19V-DNA and IgM without IgG indicated acute infection. The presence of IgG and B19V DNA in the absence of IgM indicated persistent infection. Lastly, the presence of IgM and IgG ELISA and B19V DNA indicated reactivated infection [47].

### 2.6. Amplification of the NS1-VP1u Region by Semi-Nested PCR

B19V genotyping was performed using a semi-nested PCR targeting the NS1-VP1u gene as previously described [14]. Briefly, in the first round of amplification, the primers PVB1 (5′-CACTATGAAAACTGGGCAATAAAC-3′) and B19SR (5′-CCAGGCTTGTGTAAGTCTTC-3′) were used to amplify a DNA fragment of 944 bp covering almost the entire NS1-VP1u gene. In the second round of amplification, the primers PVB3 (5′-ATAAACTACACTTTTGATTTCCCTG-3′) and B19SR were used to amplify a 926 bp fragment which identified the B19V Genotypes 1, 2 and 3. A semi-nested PCR mixture included 5 µL of DNA for Round 1, 12.5 µL of GoTaq Green Master Mix (Promega, Madison, Wisconsin, USA), 0.5 µM of both sense and antisense primers and sterile nuclease-free water (total reaction: 25 µL). PCR conditions were as follows: 3 min of an initial denaturation step at 95 °C, followed by 35 cycles of 45 s of denaturation at 95 °C, 45 s of annealing at 58 °C, and 1 min at 72 °C for the extension. The second PCR round’s parameters were the same as in the first round except for the template volume (1 µL) and the cycle number (30 cycles). Final extensions at 72 °C for 5 min were performed in both rounds. The amplified PCR fragments (926 bp) were visualized on a 2.0% agarose gel and stained with GreenView Plus Nucleic Acid gel stain. The positive bands were excised and processed further for Sanger sequencing using the AB13730XL sequencer (Applied Biosystems, Foster City, CA, USA).

### 2.7. Sanger Sequencing of the NS1-VP1u Region

All positive samples were sequenced in both directions with primers tagged with M13 using the dideoxynucleotide chain terminator method (Taq Dye Deoxy Terminator Cycle Sequencing Kit, Applied Biosystems GmbH, Weiterstadt, Germany). Sequenced forward and reverse strands were assembled using Seqman Pro and edited by EditSeq modules (DNASTAR Lasergene Sequence Analysis Software version 17.1, Madison, WI, USA). To confirm positive results, consensus sequences were blasted against the NCBI database.

### 2.8. Phylogenetic Analysis

Sequences were aligned using CLC Sequence Viewer (version 8.1.1 Aarhus, Denmark) and the phylogenetic tree was generated using the neighbor-joining algorithm based on the Kimura two-parameter distance estimation method as previously described [15,48]. Bootstrapping and reconstruction were carried out with 1000 replicates to obtain the confidence level of the phylogenetic tree. For the NS1-VP1u gene nucleotide sequence homology comparison, sequences were compared with reference sequences representing the three main B19V genotypes from the GenBank database: Genotype 1A: PVBAUA prototype (M13178.1), Wi strain (M24682) [11,49], NC_000883, DQ225150, DQ225151, DQ408301, Z68146, DQ225149, AF162273, and AY504945; Genotype 1B: DQ357065 and DQ357064 [14]; Genotype 2: Lali prototype (AY044266) and A6 strain (AY064475) [10,50]; Genotype 3: V9 strain (AX003421) and D91.1 strain (AY083234) [51].

### 2.9. Statistical Analysis

In this study, a chi-square test was used to determine any significant differences between the percentages of variable categories. The results were considered statistically significant if the *p*-value was < 0.05. The GraphPad Prism 7.00 software was used for data and statistical analysis.

## 3. Results

### 3.1. Demographic Characteristics of the Donors

Table 2 summarizes the demographics of the studied population. In total, 5026 blood samples were analyzed in the current study, of which 4862 (96.73%) were from males, 149 (2.69%) were from females, and 15 (0.29%) were unspecified. Most of the samples were obtained from non-Qatari national residents (78.33%) and the remaining samples were from Qatari nationals (20.79%). The age of donors ranged between 19 and 89 years old.

### 3.2. B19V Viremia Rates among Healthy Blood Donors

B19V DNA was detected in 1.4% (70/526) of the tested samples (Table 3). The results showed a significant negative correlation between viremia rates and age (*p* = 0.034). B19V infection decreased with age from 2.24% in the 19–30 age group to 0.87% in the 41–50 age group. However, no significant association was observed between the viremia rate and gender or nationality. The viral load of the positive samples ranged between 1.2 × 10^1^ and 8.27 × 10^6^ genome copies/mL of blood (Table 4). The serological results and viral load for all 70 B19V qPCR–positive blood donation samples are shown in Tables S1 and S2.

### 3.3. B19V Seroprevalence and Correlation with Gender, Geographic Origin, and Age

In total, 930 selected samples were serologically tested in order to determine the seroprevalence of B19V antibodies (anti-VP1/VP2 IgM and IgG) among the healthy blood donors from different nationalities in Qatar. Of these, 627 samples (62.7%) were IgG-seropositive, while 40 (4%) samples were IgM-positive (Table 5). Associations between B19V seroprevalence and gender, geographic background, and age of the studied individuals were calculated using the chi-square test.

**Figure 1:**
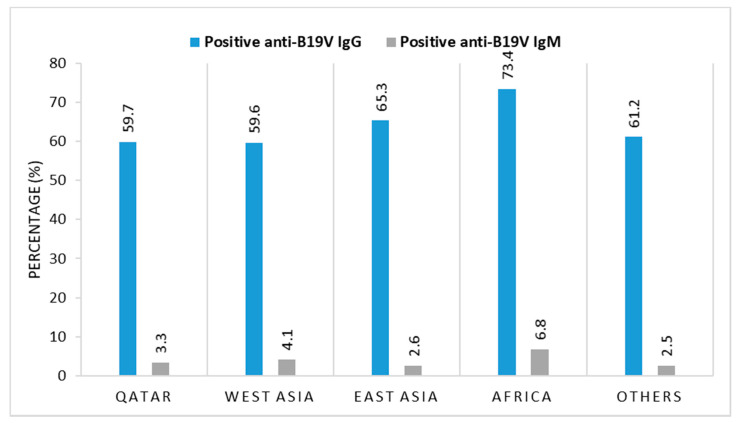
B19V IgG and IgM Seroprevalence among Major Nationalities †West Asia includes the Gulf area: KSA, Kuwait, Bahrain, Yemen, and Oman. ††East Asia includes India, Sri Lanka, Nepal, Bangladesh, Indonesia, and the Philippines.

There was a statistically significant association between B19V seroprevalence and gender (*p* = 0.044). However, this significance could be not true due to the small sample size of females compared with males. Moreover, considering that Qatar is a country with a diverse population, with expatriates constituting more than 85% of the total population, we investigated B19V frequency between Qataris (*n* = 298) and non-Qataris (*n* = 632). Specifically, we compared B19V rates among Qataris with those from three major regions: West Asia (*n* = 270), East Asia (*n* = 150), and Africa (*n* = 132) (Figure 1). There was no significant difference between Qataris and non-Qataris (*p*= 0.63) in B19V seroprevalence, where 63.1% Qatari donors were seropositive compared with 62.2% seropositivity among donors from other geographic regions. Similarly, the correlation between B19V seroconversion and the age of donors was investigated. Moreover, we found that infection rates did not increase with age (*p* = 0.919); seropositivity ranged from 60.5% in donors less than 30 years old to 63.1% in donors above 50 years of age (Table 5), suggesting that most people acquire the infection and develop antibodies at early ages.

### 3.4. Identifying the B19V Immune Status of the Donors

The immune status of the 70 positive B19V DNA samples was determined based on IgM and IgG ELISA, and the B19V-DNA results. Infection stage was assigned as: reactivated, persistent, or acute infection, according to the criteria summarized in Table 1. Of the 70 tested samples, 5.7% were in the acute/recent infection stage, 28.6% were in the reactivation stage, and 65.7% had a pattern of persistent infection (Table 4). B19V DNA viral load in each category varied in quantity, ranging between 1.2 × 10^1^ and 8.27 × 10^6^ DNA copies/mL.

### 3.5. Circulating B19V Genotypes among Blood Donors

**Figure 2:**
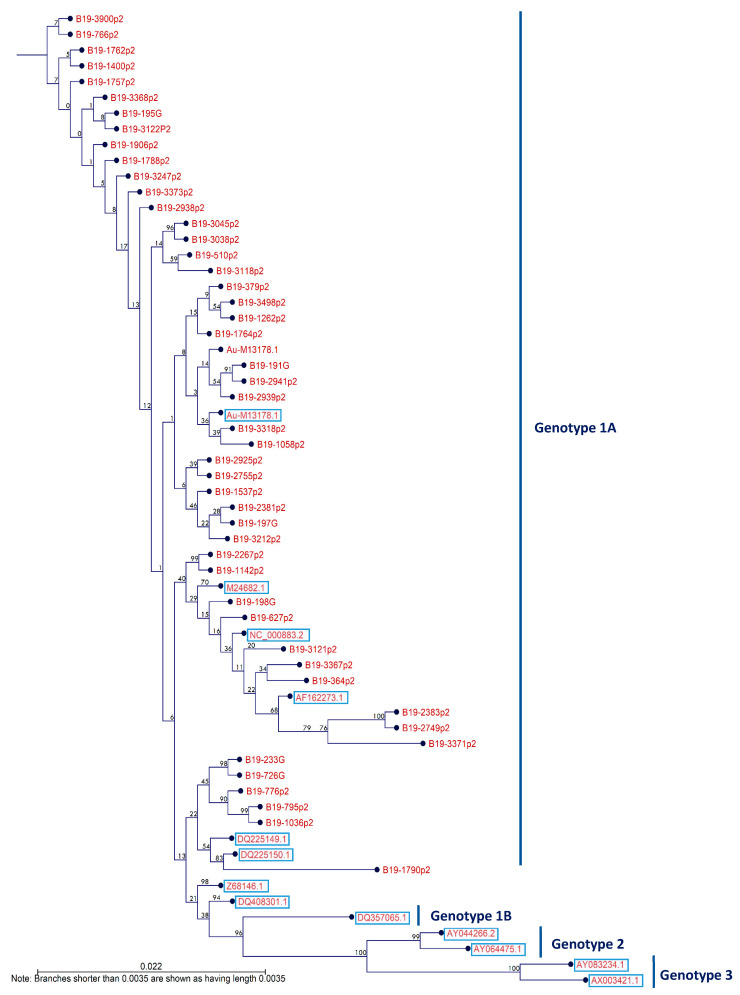
Phylogenetic tree of B19V based on the NS1-VP1u region using the neighbor-joining method and the Kimura 2 parameter model. The reference sequences are shown inside the boxes and are labeled with their GenBank accession numbers. Bootstraps values of 1000 replicates are indicated above the nodes. Branch lengths are drawn to scale.

In total, 70 samples which were positive for B19V DNA were further subjected for genotyping using the NS1-VP1u gene. Of these, 22 were not detected by nested PCR and therefore were excluded from the subsequent sequencing. In all 48 samples which were successfully genotyped, B19V Genotype 1 was predominant (100%) in both Qatari and non-Qatari participants. None of the samples was positive for either Type 2 or 3 (Figure 2). A phylogenetic tree based on 48 sequences from the current study and 16 reference sequences from GenBank revealed the presence of B19V Genotype 1 (48 of 48 (100%)); more specifically, genotype 1A. Phylogenetic analysis was based on 927 bp of a NS1-VP1u fragment (nucleotide positions 1765 to 2692) (Figure 2). More precisely, the nucleotide sequence of the 48 blood donors displayed a higher degree of similarity relative to the reference sequence of the Au strain. A summary of all the reported sequence variations is rep-resented in supplementary data (Tables S3 and S4, Figures S1 and S2).

One hundred and thirty-nine nucleotide mutations were found in the sequences of 24 samples. The most frequent change was at 2453 nt, where eight samples had an A to G nucleotide mutation that differed from the standard Au strain. This was followed by alterations at 2268, 2352, and 2531 nt, each of which was found in five samples (Table 6). The most frequent polymorphism observed were inversions (A/G, 30 positions; C/T, 28 positions) in comparison with the reference strain. Of interest, the highest rate of mutations was observed in sample No. 1790, which possessed 40 nucleotide substitutions.

## 4. Discussion

B19V is known to cause erythema infectiosum and is one of the blood-borne infectious agents that could be transmitted through infected blood donated by asymptomatic blood donors. Studies proposed that chronic carrier status of B19V is perhaps more frequent than initially thought [52,53]. Additionally, B19V viremia may not be accompanied by symptoms of infection, thus increasing the potential of transmitting infected blood or blood products to the recipient within this window period [54,55].

The clinical progression following B19V infection would differ according to the patient’s immune status and the presence of anti-B19V neutralizing IgG. Considering its importance for high-risk populations, several blood donation organizations and blood product manufacturers have implemented screening procedures to detect B19V DNA by PCR using mini-pools, such as in Germany, Austria, Poland, and Japan [33,56,57]. Although B19V may cause serious complications, donor screening for this virus is not yet mandatory in Qatar. Few studies in the Middle East and North Africa (MENA) region have described the sero-epidemiological profile of the virus; however, none has been conducted in Qatar. In the present study, we reported the seroprevalence of B19V and the circulating virus genotypes among blood donors in Qatar.

The study revealed a B19V IgG seroprevalence of 59.7% among Qatari and 60.6% among non-Qatari blood donors. This seroprevalence is comparable with previous estimates reported worldwide and in the MENA region. For instance, two studies in Tunisia and Sudan reported a B19V IgG seroprevalence of 65% and 63.3% in blood donors, respectively [58,59]. In North India, a study of volunteer blood donors reported a seroprevalence of 61.8% of B19V IgG antibodies [60]. Similarly, other studies conducted among Dutch, Brazilian, Spanish, and Korean blood donors showed IgG seroprevalence rates of 60.9%, 60%, 64.7%, and 60.1%, respectively [34,61,62,63,64]. Our B19V IgG antibody seroprevalence in blood donors was lower compared with those in Belgium (74%) [58], Makkah (76.3%) [65], and Italy (79.1%) [66]. Conversely, seroprevalence was higher than in studies of blood donors in India (39.97%) [67], China (16.8%) [68], Egypt (26%) [59], Yemen (46%), Iran (27.6%) [69], and Pune (27.9%) [60]. This could be due to the variations in age distribution among the study groups, socioeconomic status, the size and demographic characteristics of the study populations, the diagnostic techniques, and seasonal timing differences, since the infection peaks predominantly during late winter and early spring [70]. Moreover, the B19V-specific IgG is detectable about 15 days after the onset of infection, remains elevated for several months, and persists for an extended period, indicating lifelong immunity [67].

Overall, there was a significant difference (*p* = 0.0449) in B19V IgG seroprevalence between male and female blood donors. Similarly, various studies performed in Taiwan, the United States, Brazil, and Sudan, showed gender differences in the prevalence [59,71,72,73]. In contrast, other studies conducted in Brazil and Australia showed no gender differences in the prevalence [74,75]. The difference between age groups was expected, as previous studies showed that most people acquire antibodies against B19V before the age of 15 years and it increases in prevalence with age. This implies that B19V is age-dependent, and that throughout life, new infections arise, leading to a continuous increase in seroprevalence up to more than 80% in the elderly (>70 years) [62]. However, in our study, only a slight and statistically insignificant increase in the B19V seroprevalence with age was observed. This could be attributed to the fact that our study population was mostly composed of expatriates from the same age group. The lack of correlation between B19V seroprevalence and age has also been reported from studies in Egypt [76], Nigeria [77] and Sudan [59]; however, all these studies had relatively small sample sizes (≤ 180). We observed comparable seropositivity rates of B19V IgG among different age groups, with the 31–40 age group exhibiting the lowest seropositivity rate (60.6%). This aligns with the figures of Turkish blood donor groups, where the 18–30- and 41–50-year-old groups showed 60.3% and 64.7% seroprevalences, respectively [78]. Indeed, Iran, Canada, and a population-based study in Amsterdam revealed similar progressively persistent prevalence rates in different age groups [69,79,80]. In Saudi Arabia, a country bordering Qatar, the B19V infection was 69.8% by the age of 39 [65]. By comparison, seroprevalence rates of 64.8% and 64.0% in the two age ranges of 18–34 and 35–44 were found in Amsterdam [80].

Traditionally, the diagnosis of B19V infection relies on the detection of specific antibodies using ELISA due to its high sensitivity [81]. However, estimating the viral DNA load in blood assists in the staging of the infection (e.g., acute, past, or reactive stage), especially in pregnant women, and immunosuppressed or immunocompetent individuals [82]. Therefore, the combination of quantitative molecular PCR and serology is increasingly recommended for B19V diagnosis to differentiate recent from past infections [83,84,85]. In this study, B19V DNA was detected in 1.4% of donors (70/5026) using qPCR, suggesting the presence of recent B19V infection at the time of donation. Additionally, IgM antibody was detected in 20 (28.6%) of B19V DNA-positive donors. This might be because the blood donation occurred prior to seroconversion, giving negative IgM antibody results, or occurred after IgM antibody levels began to decline [86]. Another explanation is the possible high-level viremia in acutely infected cases, which creates virus–antibody complexes that result in false negative IgM findings [87]. This suggests that PCR may be a better diagnostic tool in such situations. Similar viremia rates were reported in some developing countries like Brazil (1%), Ghana (1.3%), Malawi (1.25%), and Iran (1.2%) [17,63,69]. On the other hand, low rates of B19V infection have been reported in immunocompetent blood donors in developed countries. In two studies targeting US blood donors, viremia rates of 0.1% and 0.88% were reported [52,82]. In European countries, the reported viremia rates among blood donors were 0.013% and 0.26% in Germany and Austria [33], 0.03% in the UK [27], 0.12% in Portugal [88], 0.16% in Belgium [35], and 0.006% in the Netherlands [61]. In addition to 0.6% in Japan [36]. The seemingly high viremia rates in the current study might be expected due to the highly diverse population in Qatar.

Although different B19V genotypes have been described based on the NSI-VP1u region of the B19V virus [14,16,17,89], sequence analysis has not allowed the identification of phylogenetic clusters with well-resolved nodes within the B19V viruses [90,91]. In two different studies, it was reported that nested PCR targeting the NS1-VP1u region failed to detect V9 DNA for D91.1 and the Lali strain due to primer mismatch. This was observed in 100 B19V IgM-positive serum samples or plasma pools from 100,000 Danish blood donor units, and in four of 11 in the French population [1,92]. This suggests that PCR assays may fail to reliably detect all B19 genotypes [80]; hence, it may explain, to some extent, why the NS1-VP1u PCR products for 22 of the 70 B19V-positive donors was not successfully obtained in the current study. Indeed, we may have V9-related sequences in the MENA region, which can be further investigated in future studies. Moreover, while weak B19V amplification can still be detected by qPCR, identification of the viral genotype by nested PCR and sequencing of the partial NS1-VP1u genomic region could be missed due to low viral load [93]. Additionally, all nested PCR negative samples in this study were persistent or reactivated infections, and a relationship between persistence and a high degree of genetic variability was previously reported [94,95].

The prevalence and genetic diversity of B19V genotypes was correlated with population, geographic origin, the time of collection, and sample type (blood/tissue) [96,97]. Interestingly, our study revealed that Genotype 1 was predominant (100%) across different nationalities, based on the partial NS1-VP1u junction’s sequence; all isolates detected were classified as Sub-genotype 1A. Although infection with one genotype does not provide immunity against the other genotypes [98], none of the tested isolates were Genotype 2 or 3 or had co-infection, in contrast to what has been previously reported in other studies [99]. Similar findings were reported among Iranian and Dutch blood donors, in which all of the donors were B19V Genotype 1A [69,100]. This genotype is circulating worldwide (78%) and causes most of the infections, compared with Genotype 2, which is more prevalent in Europe, Vietnam, and Brazil [10,45,83,101]. Genotype 3 has been detected in French and Brazilian patients [1,102] and Ghanaian blood donors, the endemic regions for that genotype [17,92,101]. The current results are in agreement with those of Heegaard et al. and suggest that viremic infections by the new genotypes, at present, are rare [101].

NS1 protein is crucial for viral replication and packaging [103]. Thus, any mutations in NS1 could lead to the pathogenicity of B19V infection through modulation of its cytotoxicity [90,104,105]. This may explain why NS1 is highly conserved [104,106]. On the other hand, VP1u is the most variable region in the genome of B19V [90]. The NS1-VP1u region covers nearly one-fifth of the viral genome and includes genetic information on three viral proteins (NS1, VP1u, and a 7.5-kDa protein), spanning two recognized dominant neutralizing epitopes [1]; hence, it is appropriate for phylogenetic analysis. Accordingly, the NS1-VP1u region was chosen in the present study to make phylogenetic comparisons against the reference sequences (Figure 1).

Analysis of the single nucleotide mutations in each sample revealed the presence of 139 nucleotide mutations in the sequences of 24 samples. The most frequent change was at 2453 nt, which was detected in eight samples. This was followed by mutations at positions 2268, 2352, and 2531, each of which was found in five samples. Interestingly, one of the samples had 40 nucleotide substitutions, which may explain the long branch seen in the phylogenetic tree. Moreover, while some sequence samples clustered reliably with the reference sequence, some of our sequences clustered with each other.It was previously reported that B19V genotypes and subtypes have a high ratio of synonymous to nonsynonymous nucleotide changes per site, suggesting that the NS1 region is under strong purifying selection [1,91]. Therefore, most of our samples exhibited changes in only few positions in their DNA sequence but not in the protein sequence due to silent mutations. The relation between high genetic diversity and low amino acid variability observed in human parvovirus is consistent with the apparent lack of difference in clinical manifestations, pathogenicity, and antigenic reactivity among genotypes [1,16,95,107,108,109,110]. Therefore, the three genotypes have the same structural, functional, and immunological characteristics and comprise the same serotype [111].

This study has some limitations, including the fact that samples from the same repetitive blood donors cannot be identified and excluded. However, we see no reason to postulate that the donation patterns of B19V-seropositive donors are different from those of B19V-seronegative donors. Another limitation is the lack of information about some potential risk factors, such as the period of residency in Qatar, travel history, transfusion history, if participants have any children, and date of sample collection. Such information was not available, since limited information concerning the characteristics of the donors was collected. Moreover, most of the donors in this study were males, representing 96.73% of the total donors screened. Since they are the major donors, our estimates involve a limitation in the representativeness of the findings and may not fully reflect that of all Qatar, which may bias the comparison between the two groups. Similar observations regarding this gender bias were previously reported in India, Nigeria, Zambia, and Japan [60,77,112,113]. Furthermore, we have not considered seasonality and periodicity in the model, since the infection peaks predominantly during the months of late winter and early spring [70]. Finally, small errors in antigenic testing results, e.g., nonspecific false positive results, cannot be ruled out [113]. The study was limited to samples collected from donors above 18 years of age, and since most people become infected with B19V before the age of 15 years old, a similar study with younger groups is recommended, especially when investigating B19V seroconversion and transmission.

## 5. Conclusions

To summarize, this is the first study to investigate the seroprevalence and molecular epidemiology of B19V in Qatar. The study cohort revealed the predominance of only Genotype 1A. Moreover, the seroprevalence of B19V among the blood donor population in our study was high, suggesting endemicity of infection. The identification of 1.4% of blood donations containing B19 DNA might hypothetically pose a potential risk, especially for high-risk blood product recipients. Therefore, it is recommended to administer safe cellular blood products to all high-risk patients [67,85]. Accordingly, mini-pool whole blood screening should be planned in blood banks and organ transplant centers. The screening is already applicable in several countries around the world as an in-process control to ensure that levels of B19V DNA do not exceed 5 × 10^6^ copies/mL in the manufacturing pool [114], or the blood units have to be IgM- and PCR-negative. This will contribute to decreasing not only the viral load in pooled source plasma but also the frequency of seroconversion or symptomatic infection after treatment with blood products. Further studies will be required to elucidate the biological significance of the unclassified strains [48].

## Figures and Tables

**Table 1:** Interpretation of human parvovirus (B19V) serological patterns in immunocompetent individuals.

Interpretation	B19V Marker		
	DNA	IgM	IgG
Acute infection	+	+/−	−
Persistent	+	−	+
Reactivated	+	+	+

**Table 2:** Demographic characteristics of the study sample (*n* = 5026).

	Characteristic	No (%)
Sex		
	Male	4862 (96.73)
	Female	149 (2.96)
	Unknown	15 (0.29)
	Total	5026
Age (years)		
	19–30	1124 (22.36)
	30–40	2061 (41.00)
	40–50	1275 (25.36)
	>50	543 (10.84)
	Unknown	23
	Total	5026
Nationality		
	Qatari	1045 (21)
	Non-Qatari	3981 (79)
	Egyptian	827 (16.4)
	Sudanese	112 (2.2)
	Philippine	115 (2.3)
	Indian	555 (11)
	Sri Lankan	77 (1.5)
	Lebanese	108 (2.1)
	Jordanian	311 (6.2)
	Palestinian	271 (5.4)
	Syrian	644 (12.8)
	Pakistan	197 (3.9)
	Iranian	99 (2)
	Yemen	124 (2.5)
	Other *	541 (11)
	Total	5026

* “Other” included Kuwait, United Arab Emirates (UAE), Kingdom of Saudi Arabia (KSA), Oman, Bahrain, Iraq, Morocco, Algeria, Libya, Tunisia, Turkey, Bosnia, Cyprus, Greece, Russia, Malaysia, Bangladesh, Nepal, Burma, Singapore, USA, Canada, Spain, Bulgaria, Ireland, UK, Kenya, Somalia, France, Italy, Romania, Germany, Colombia, Brazil, New Zealand, Hungary, the Netherlands, Croatia, Ecuador, Serbia, Macedonia, Sweden, Australia, South Africa, Eritrea, Burkina Faso, Djibouti, Chad, Tanzania, and Ethiopia.

**Table 3:** B19V viremia in the studied population (*n* = 5026).

Category	Total No.	B19V DNA qPCR Positive No. (%)	*p*-Value *
Gender			
Male	4862	68 (1.4)	0.453
Female	149	1 (0.7)	
Unknown	15	1 (6.7)	
Nationality			
Qatari	1045	16 (1.5)	
Male	1007	15 (1.5)	
Female	37	0 (0.0)	
Non-Qatari	3937	49 (1.2)	0.468
Male	3825	48 (1.3)	
Female	111	1 (0.9)	
Unknown	44	1 (2.3)	
Age Group			
19–30	1124	25 (2.2)	
31–40	2061	26 (1.3)	0.034
41–50	1275	11 (0.9)	
> 51	543	7 (1.3)	
Unknown	23	1 (4.4)	

* Pearson’s Chi-Square *p*-value; unknown samples were excluded from the statistical analysis.

**Table 4:** B19 infection stages according to ELISA and qPCR (*n* = 70).

Category by ELISA	No. (%)	Viral Load Copies/mL of Blood
Acute infection	4 (5.7)	-
Reactivated	20 (28.6)	1.2 × 10^1^–5.95 × 10^6^
Persistent	46 (65.7)	7.41 × 10^1^–8.27 × 10^6^
Total	70	

**Table 5:** B19V seroprevalence in the studied population (*n* = 930).

Category	Total No.	B19V Serology			
		IgG-Positive No. (%)	*p*-Value *	IgM-Positive No. (%)	*p*-Value *
**Gender**					
Male	808	495 (61.2)	0.0449	17 (2.1)	0.649
Female	122	66 (54.1)		3 (2.4)	
Total	930	561 (60.3)		20 (2.1)	
**Nationality**					
Qatari	298	178 (59.7)	0.6335	10 (3.3)	0.648
Male	261	158 (60.5)		9 (3.4)	
Female	37	20 (54.1)		1 (2.7)	
Non-Qatari	632	383 (60.6)		10 (1.6)	
Male	547	337 (61.6)		8 (1.4)	
Female	85	46 (54.1)		2 (2.3)	
**Age Group**					
19–30	307	182 (59.3)	0.9194	4 (1.3)	0.2132
31–40	335	203 (60.6)		7 (2.1)	
41–50	209	127 (60.7)		9 (4.3)	
> 51	76	48 (63.1)		0 (0)	
Unknown	3	1 (33.3)		0 (0)	

* Pearson’s Chi *p*-value.

**Table 6:** Comparisons between different B19V samples at the DNA and protein levels.

nt Position	nt Change	Amino Acid Codon	Amino Acid Change	No of Samples with Mutation
1926	C/G	1926	R > T	1
1929	C/T	1929	F > S	2
1930	A/C/T	1932	K > Q	3
1939	A/C	1941	Q > p, Q > S	2
1967	G/C	1968	A > P	1
1997	G/C	1998	V > L	1
2036	G/A	2037	G > N, G > R	1
2235	T/C	2235	V > A	2
2244	A/G	2244	N > H, N > C	3
2268	G/C	2268	G > A	5
2309	A/G	2310	I > V	1
2352	A/G	2352	D > G	5
2453	A/G	2454	K > E	8
2531	G/C	2532	V > L	5
2548	G/A	2550	E > K	1
2555	A/G	2556	I > K, I > V	1
2603	G/A	2604	E > P, E > K	1
2616	C/T	2616	S > L	1
2620	G/A	2622	F < L	1
2629	G /A	2631	V < I	1
2646	A/T	2646	N > I	1

## Data Availability

Not applicable.

## Supplementary Materials

The following are available online at https://www.mdpi.com/1999-4915/13/4/540/s1.

## Abbreviation

B19	Human parvovirus (B19)
EDTA	Ethylenediamine tetraacetic acid
ELISA	Enzyme-linked immunosorbent assay
HMC	Hamad Medical Corporation
IRB	Institutional review board
MENA	Middle East and North Africa
qQPCR	Quantitave real-time polymerase chain reaction
NS	Nonstructural protein
