# Supplementary material for: Prevalence and Phylogenetic Analysis of Parvovirus (B19V) among Blood Donors with Different Nationalities Residing in Qatar
Source: Viruses. 2021 Mar 24;13(4):540. doi: 10.3390/v13040540 (PMC8063948; doi:10.3390/v13040540)
Supplement: Supplementary file 1 [file viruses-13-00540-s001.pdf]

Supplementary data

Table S1. Serological results for all 70 B19V qPCR–positive blood donation samples in Qatar

Sample label	Sex	Nationality	Age	IgG result	IgM result	PCR results
263 p2	Male	Egypt	35	+	+	(+)
2267 P2	Male	Egypt	35	+	+	(+)
2939 P2	Male	Egypt	39	+	-	(+)
3038 P2	Male	Egypt	54	+	+	(+)
3122 P2	Male	Egypt	28	+	+	(+)
3210 P2	Male	Egypt	28	+	+	(+)
3118 P2	Male	Egypt	27	+	-	(+)
3498 P2	Male	Egypt	40	+	+	(+)
3903 P2	Male	Egypt	36	+	-	(+)
766 p2	Male	India	31	-	-	(+)
1554 P2	Male	India	30	+	-	(+)
1757 P2	Male	India	24	+	-	(+)
1906 P2	Male	India	35	+	+	(+)
3045 P2	Male	India	38	+	-	(+)
652P1	Male	India	24	Not tested	Not tested	(+)
3057 P2	Male	India	26	+	-	(+)
2383 P2	Male	Iran	55	+	-	(+)
3373 P2	Male	Iran	21	+	-	(+)
195 P1	Male	Iran	44	-	-	(+)
510 P1	Male	Jordan	35	+	-	(+)
2765 P2	Male	Jordan	35	+	-	(+)
2938 P2	Male	Jordan	40	+	+	(+)
3121 P2	Male	Jordan	40	+	+	(+)
3247 P2	Male	Jordan	44	+	-	(+)
3367 P2	Male	Jordan	44	+	-	(+)
1036 p2	Male	Lebanon	35	+	-	(+)
348 P1	Male	Nepal	28	+	+	(+)
726 P1	Female	Palestine	37	+	+	(+)
1142 P2	Male	Palestine	37	+	+	(+)
2381 P2	Male	Palestine	29	+	+	(+)
3318 P2	Male	Palestine	59	+	-	(+)
198 P1	Male	Palestine	47	+	-	(+)
379 P1	Male	Philippine	27	+	-	(+)
3212 P2	Male	Philippine	28	+	+	(+)
3213 P2	Male	Philippine	34	+	-	(+)
359 P2	Male	Qatar	34	+	-	(+)
364P2	Male	Qatar	29	+	-	(+)
1058 p2	Male	Qatar	31	+	-	(+)
1262 P2	Male	Qatar	46	+	-	(+)
1400 P2	Male	Qatar	45	+	+	(+)

1537 P2	Male	Qatar	34	+	-	(+)
1556 P2	Male	Qatar	20	+	-	(+)
1788 P2	Male	Qatar	31	+	-	(+)
2386 P2	Male	Qatar	35	+	-	(+)
3368 P2	Male	Qatar	39	+	-	(+)
3321 P2	Male	Qatar	29	+	-	(+)
3900 P2	Male	Qatar	32	-	-	(+)
191 P1	Male	Qatar	64	+	-	(+)
197 P1	Male	Qatar	41	+	-	(+)
756 P1	Male	Qatar	33	Not tested	Not tested	(+)
233 P1	Male	Qatar	44	+	-	(+)
772 p2	Male	Sudan	58	+	-	(+)
2749 P2	Male	Sudan	41	+	+	(+)
627 p2	Male	Syria	43	+	-	(+)
795 p2	Male	Syria	29	+	+	(+)
1762 P2	Male	Syria	34	+	-	(+)
2777 P2	Male	Syria	29	+	-	(+)
2755 P2	Male	Syria	62	+	-	(+)
2941 P2	Male	Syria	24	+	+	(+)
3209 P2	Male	Syria	29	+	-	(+)
3214 P2	Male	Syria	34	+	+	(+)
3371 P2	Male	Syria	53	+	-	(+)
645 P1	Male	Syria	23	Not tested	Not tested	(+)
1790 P2	Male	United Kingdom	40	+	-	(+)
2945 P2	Unknown	Unknown	Unknown	+	-	(+)
588 P1	Male	Yemen	47	+	+	(+)
1764 P2	Male	Yemen	23	+	-	(+)
2946 P2	Male	Yemen	24	+	-	(+)
643 P1	Male	Pakistan	26	+	-	(+)
977 P1	Male	Morocco	35	-	-	(+)

Table S2. Comparison between DNA positive donors and other tests

Donor ID	B19V DNA		Anti-B19V		
	QPCR	Viral load	IgG	IgM	Infection status
726	Positive	4.29×10^5	Positive	Positive	Reactivated
348	Positive	3.61×10^5	Positive	Positive	Reactivated
588	Positive	4.79×10^6	Positive	Positive	Reactivated
263	Positive	2.14×10^4	Positive	Positive	Reactivated
359	Positive	7.41×10^1	Positive	Negative	Persistence
364	Positive	3.15×10^3	Positive	Negative	Persistence
379	Positive	2.91×10^3	Positive	Negative	Persistence
510	Positive	4.11×10^4	Positive	Negative	Persistence
627	Positive	Need to be confirmed	Positive	Negative	Persistence
766	Positive	2.71×10^3	Negative	Negative	Acute/Recent
772	Positive	2.88×10^5	Positive	Negative	Persistence
795	Positive	Need to be confirmed	Positive	Positive	Reactivated
1036	Positive	7.73×10^5	Positive	Negative	Persistence
1058	Positive	Need to be confirmed	Positive	Negative	Persistence
1142	Positive	Need to be confirmed	Positive	Positive	Reactivated
1262	Positive	1.46×10^4	Positive	Negative	Persistence
1400	Positive	1.36×10^6	Positive	Positive	Reactivated
1537	Positive	Need to be confirmed	Positive	Negative	Persistence
1554	Positive	7.61×10^5	Positive	Negative	Persistence
1556	Positive	1.95×10^6	Positive	Negative	Persistence
1757	Positive	3.85×10^5	Positive	Negative	Persistence
1762	Positive	2.04×10^6	Positive	Negative	Persistence
1764	Positive	9.37×10^2	Positive	Negative	Persistence
1788	Positive	3.11×10^6	Positive	Negative	Persistence
1790	Positive	4.16×10^6	Positive	Negative	Persistence
1906	Positive	2.39×10^4	Positive	Positive	Reactivated
2267	Positive	7.61×10^5	Positive	Positive	Reactivated
2777	Positive	5.90×10^2	Positive	Negative	Persistence
2381	Positive	3.62×10^6	Positive	Positive	Reactivated
2383	Positive	1.61×10^3	Positive	Negative	Persistence
2386	Positive	1.59×10^3	Positive	Negative	Persistence
2749	Positive	5.27×10^6	Positive	Positive	Reactivated
2755	Positive	3.85×10^6	Positive	Negative	Persistence
2765	Positive	1.18×10^5	Positive	Negative	Persistence
2938	Positive	1.40×10^6	Positive	Positive	Reactivated
2939	Positive	2.54×10^6	Positive	Negative	Persistence
2941	Positive	3.38×10^3	Positive	Positive	Reactivated
2945	Positive	8.27×10^6	Positive	Negative	Persistence
2946	Positive	5.74×10^5	Positive	Negative	Persistence

3038	Positive	1.11×10^6	Positive	Positive	Reactivated
3045	Positive	7.34×10^6	Positive	Negative	Persistence
3121	Positive	6.55×10^5	Positive	Positive	Reactivated
3122	Positive	5.95×10^6	Positive	Equivocal	Reactivated
3209	Positive	4.10×10^1	Positive	Negative	Persistence
3210	Positive	1.24×10^4	Positive	Positive	Reactivated
3212	Positive	1.2×10^1	Positive	Positive	Reactivated
3213	Positive	1.82×10^3	Positive	Negative	Persistence
3214	Positive	6.69×10^2	Positive	Positive	Reactivated
3247	Positive	9.25×10^4	Positive	Negative	Persistence
3318	Positive	6.00×10^1	Positive	Negative	Persistence
3118	Positive	1.12×10^3	Positive	Negative	Persistence
3367	Positive	4.95×10^6	Positive	Negative	Persistence
3368	Positive	3.1×10^1	Positive	Negative	Persistence
3371	Positive	6.69×10^6	Positive	Negative	Persistence
3373	Positive	1.10×10^4	Positive	Negative	Persistence
3321	Positive	8.32×10^4	Positive	Negative	Persistence
3498	Positive	6.19×10^4	Positive	Positive	Reactivated
3900	Positive	9.03×10^2	Negative	Negative	Acute/Recent
3903	Positive	1.49×10^4	Positive	Negative	Persistence
191	Positive	1.79×10^2	Positive	Negative	Persistence
195	Positive	8.10×10^1	Negative	Negative	Acute/Recent
197	Positive	9.90×10^1	Positive	Negative	Persistence
198	Positive	9.00×10^1	Positive	Negative	Persistence
233	Positive	4.58×10^4	Positive	Negative	Persistence
3057	Positive	Need to be confirmed	Positive	Negative	Persistence
756 P1	Positive	Need to be confirmed	Not tested	Not tested	
643 P1	Positive	Need to be confirmed	Positive	Negative	Persistence
645 P1	Positive	Need to be confirmed	Not tested	Not tested	
652P1	Positive	Need to be confirmed	Not tested	Not tested	
977 P1	Positive	Need to be confirmed	Negative	Negative	Acute/Recent
Total*	70		63	20	32

*Total number of positive results

Table S3. Nucleotide differences between B19-Au, B19-Lali in human parvovirus in bases 1874-2496

nt No	Au	Lali
1874	T	C
1907	A	C
1976	G	C
2066	T	G
1931	A	C
2253	C	T
2271	G	A
2274	A	C
2283	C	T
2289	C	T
2298	A	G
2301	G	A
2322	A	G
2337	C	T
2349	G	A
2361	C	T
2367	G	A
2392	C	T
2416	C	T
2433	C	T
2453	A/G	G
2461	C	T
2494	A	G
2496	C	A

Table S4. Variation in the nucleotide sequence of the region between nt 1769 and nt 1931 of B19 virus Au and Wi standard references

nt No	nt	Aa. No	Aa Change	Au	Wi
1769	C		T>N		
1773	A			A	
1873	A		I>V		
2042	C			-	G
2093	C		S>L		
2096	T		F>S	-	C
2123	T			-	C
2148	C			-	T
2223	T				
2226	A				
2268	G				
2283	C				
2286	T			-	C
2329	G		E>K		
2352	A			-	G
2353	G	4	E>K	A	-
2370	A			-	C
2392	C			-	T
2400	C			-	T
2412	T			-	C
2427	G			-	T
2433	C				
2453	G	4	K>E	A	-
2456	C			-	T
2459	G	6	G>S	-	-
2461	C			-	G
2480	G	13	D>N		
2504	C	21	Q>E	-	-
2515	G			-	A
2516	G	25	E>Q	-	-
2525	G	28	E>K	-	-
2527	A			A	G
2530	G				
2531	G		V>L	-	C
2539	A			-	T
2545	C			-	T
2560	A				
2578	T			-	C

2594	A	51	N>H	-	C
2618	C				
2624	G	61	D>N	-	A
2624	G			-	A
2645	T	68	N>H	-	-
2689	A				
2531/33	G/T	30	V>L	-	-

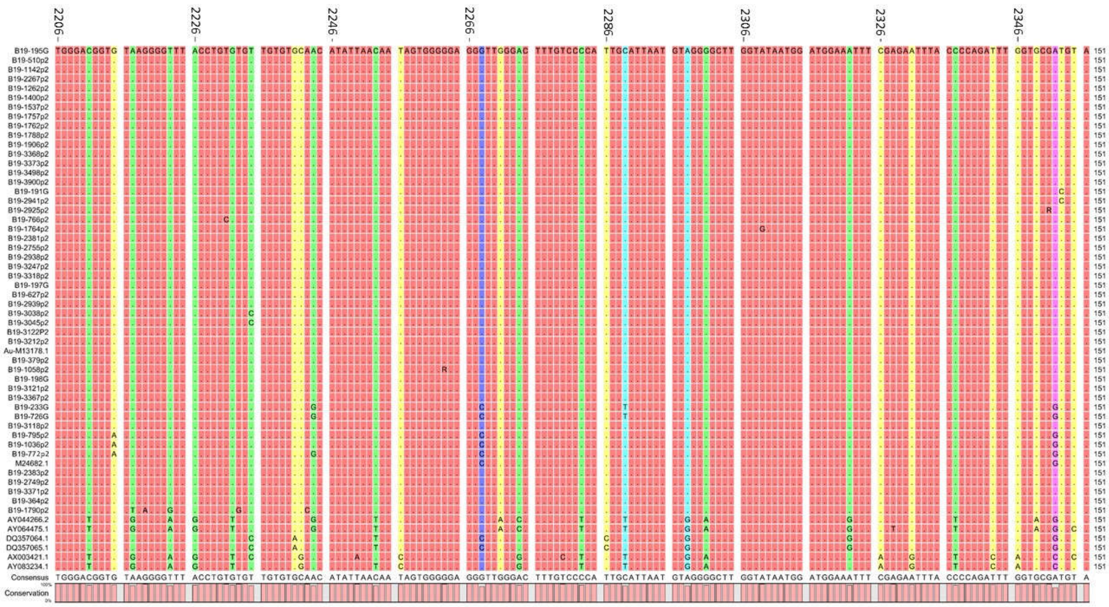

Figure 1. This figure is represents all the mutations from nt(2206-2256) in NS1VP1u junction. There was common nucleotide change in samples 233, 726, 795, 1036 and 772 at positions 2268G >C, samples 233,726 at position 2289C > T and samples 233,726, 772 at position 2244A> G, samples 795, 1036,772 at positions 2215G > A and samples 3038,3045 at position 2235T> C. There was a single nucleotide change in sample 772 at position 2231T >C, whereas sample 1970 have multiple mutation at different position 2217T>A, 2219A>G, 2223G>T, 2233G>T), 2243A>C, 2233 T>G, 2223T>G , 2042C>A. All other bases in these regions were identical. Nucleotide reading frame assignments are according to previous published papers (52, 59).

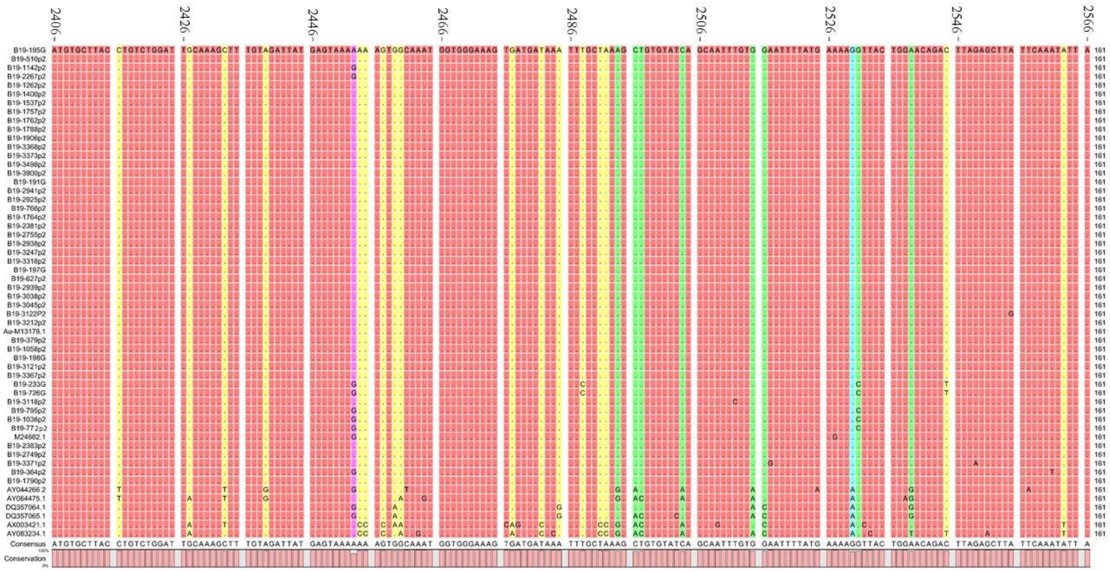

Figure 2. This figure is representing all the mutations from nt (2406–2566) in NS1VP!u junction. Sample 3212 at position 2404 A > G, samples 726, 364, 772, 795, 1036, 1142, 2267 and 233 at position 2453 A > G, samples 726, 772, 795, 1036 and 233 at position 2531 G > C, samples 726 ad 233 at position 2544 C > T, sample 3371 at position 2548 G > A and sample 3122 at positions 2544 C > A, 2555 A > G, 2561 A > T.
